# New Methodology for the Synthesis of Thiobarbiturates Mediated by Manganese(III) Acetate

**DOI:** 10.3390/molecules17044313

**Published:** 2012-04-10

**Authors:** Ahlem Bouhlel, Christophe Curti, Patrice Vanelle

**Affiliations:** Laboratoire de Pharmaco-Chimie Radicalaire, Faculté de Pharmacie, Institut de Chimie Radicalaire ICR, UMR 7273, Aix-Marseille Univ, CNRS, 27 Bd Jean Moulin, CS 30064, 13385 Marseille Cedex 05, France

**Keywords:** manganese(III) acetate, barbiturates, radical

## Abstract

A three step synthesis of various thiobarbiturate derivatives **17**–**24** was established. The first step is mediated by Mn(OAc)_3_, in order to generate a carbon-carbon bond between a terminal alkene and malonate. Derivatives **1**–**8** were obtained in moderate to good yields under mild conditions. This key step allows synthesis of a wide variety of lipophilic thiobarbiturates, which could be tested for their anticonvulsive or anesthesic potential.

## 1. Introduction

Manganese(III) acetate has been extensively explored during the past decades, and it remains an useful tool for carbon-carbon bond formation [1,2]. Its specificity to carbonyl derivatives allows a wide variety of radical synthetic applications, as studied on acetoacetate [3], β-ketoesters [4], β-ketonitriles [5,6] and β-ketosulfones [7,8,9]. Malonate derivatives, key-step substrates for barbiturates synthesis [10,11], are also useful substrates for manganese(III) acetate-mediated reactions [12,13]. In continuation of our research program centered on the design and synthesis of original molecules with pharmacological properties [14,15,16,17,18], we propose herein a manganese(III) acetate-mediated multistep synthesis of new original barbiturates.

Barbiturate derivatives are a well-known pharmacological class with anticonvulsive, sedative and anesthetic properties [19]. Original barbiturates were also recently reported as matrix metalloproteinase inhibitors with potent pharmacological applications against focal cerebral ischemia after acute stroke [20] and cancer cells invasiveness inhibitors [21]. Barbiturate derivatives also show antitubercular [22], PPAR-γ agonist [23,24,25] and protein kinase C inhibitor [26] activities. 

The lipophilicity of barbiturates is an important parameter which enhances anesthetic onset [27]. It can be improved by replacing oxygen by a sulfur [28], as seen with the very short acting barbiturate thiopenthal. Substituents on the carbons of the barbituric acid scaffold also have a great influence on the pharmacological activity [27,29]. Our methodology allows synthesis of a wide variety of substituted barbiturates, which could be tested for their anticonvulsive or anesthetic potentialities.

## 2. Results and Discussion

Starting from malonate barbiturate precursors, reproducible methodology for synthesis of various and highly functionalized derivatives was established. As reported in previously described mechanisms [30], Mn(OAc)_3_ and malonates in acetic acid form a Mn^3+^-enolate complex. Mn^3+^ is reduced in Mn^2+^, generating a carbon centered radical between carbonyl groups. This radical reacts with terminal alkene, generating a carbon-carbon bond.

**Scheme 1:**
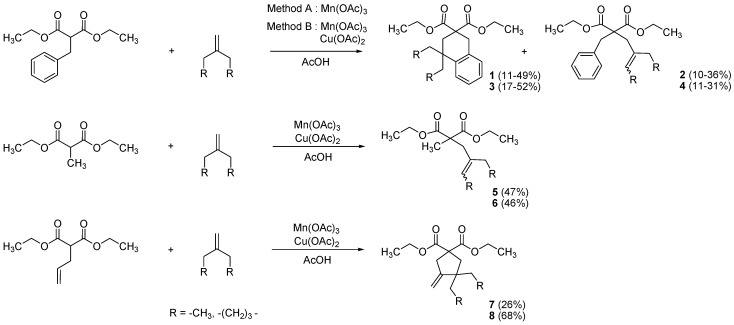
Mn(OAc)_3_ reactivity towards various malonate derivatives.

Depending on the malonate substituent, several reactions may occur and in order to investigate a larger variety of barbiturate synthesis possibilities, we have studied three of them. Results are reported in Scheme 1.

As reported by Citterio and coworkers [31,32,33], benzylmalonate allowed synthesis of two derivatives: Tetralines **1**,**3** from radical aromatic substitution, and elimination products **2**,**4**. We have previously reported different methods for optimizing yields of these two products [34]. For conditions favoring spirocyclic tetralin **1**,**3** formation, we divided up the Mn(OAc)_3_ to ensure moderate oxidizing conditions (*method A*). Tetralins **1**,**3** were obtained as the major compound (49–52%) and alkenes **2**,**4** were observed as secondary products (10–11%). Stronger oxidative conditions [Cu(OAc)_2_ + Mn(OAc)_3_, *method B*] afforded an increase in elimination products **2**,**4** (31–36%), while these conditions drastically decreased yields of tetralines **1**,**3** (11–17%).

With methyl malonate, only elimination products **5**–**6** were obtained with moderate yields (46–47%). With allyl malonate, cyclization generates a cyclopentane ring [35], and annulation products **7**–**8** were synthesized (26–68%). These three different reactivities depend on the malonate substituents, and allow access to a wide variety of substituted substrates for barbiturate synthesis.

**Scheme 2:**
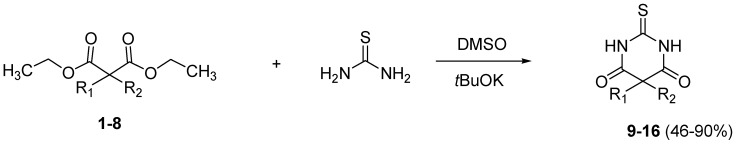
Thiobarbituric acid synthesis from malonates **1**–**8**.

*C*-Functionalized malonates **1**–**8** thus obtained reacted with thiourea [36], forming thiobarbituric scaffolds **9**–**16** in moderate to good yields (46–90%). Results are summarized in Scheme 2 and Table 1.

**Table 1:** Thiobarbituric acids **9**–**16** synthesis from malonates **1**–**8**.

Entry	R_1_,R_2_ (malonate)	Product	Yields
1	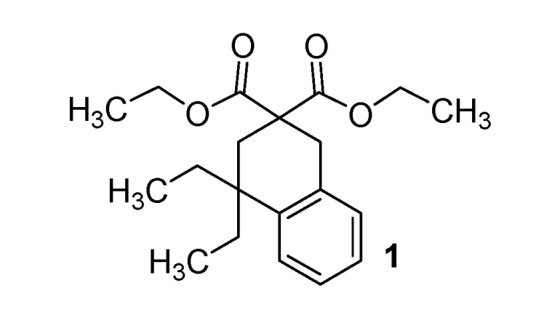	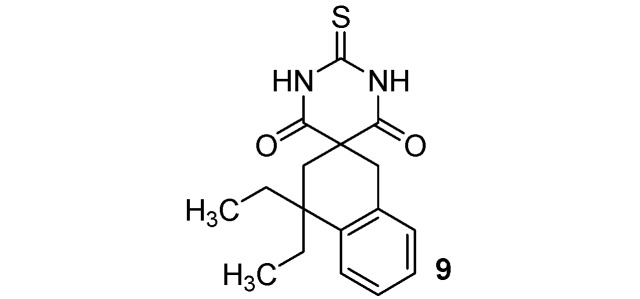	53%
2	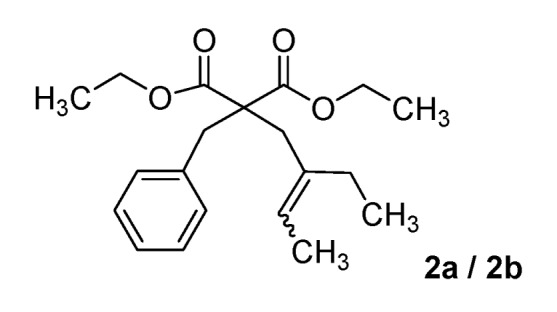	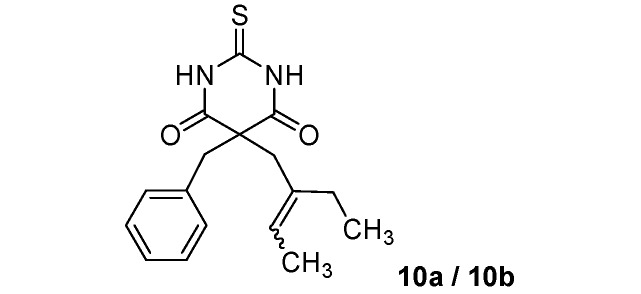	46%
3	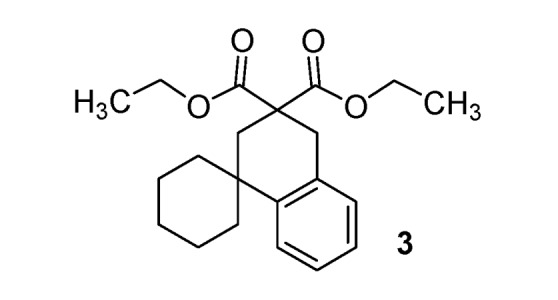	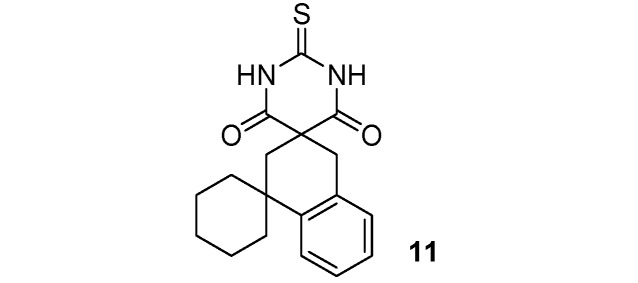	64%
4	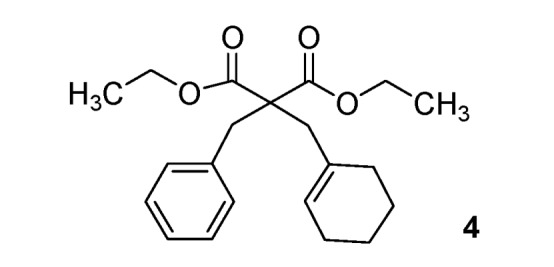	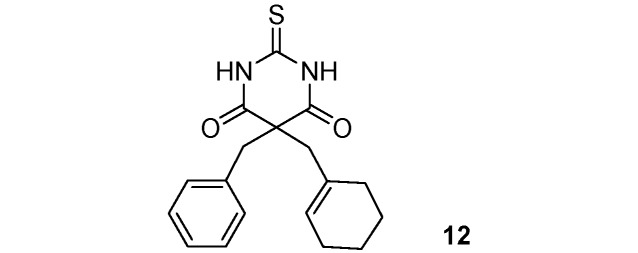	88%
5	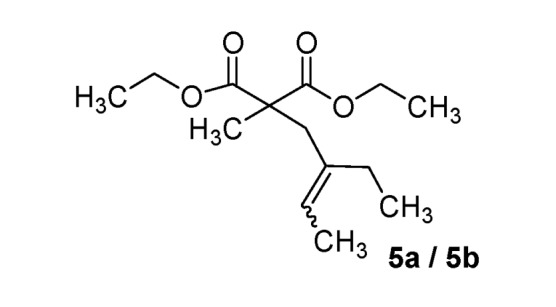	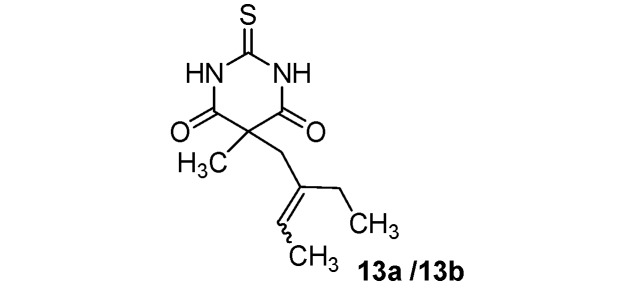	75%
6	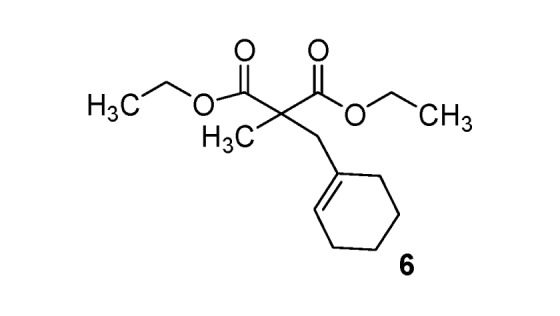	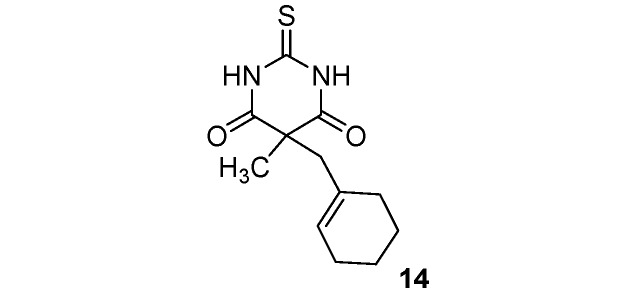	90%
7	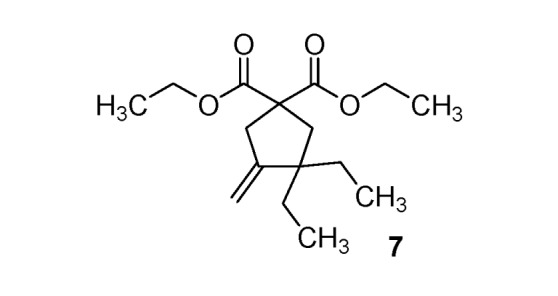	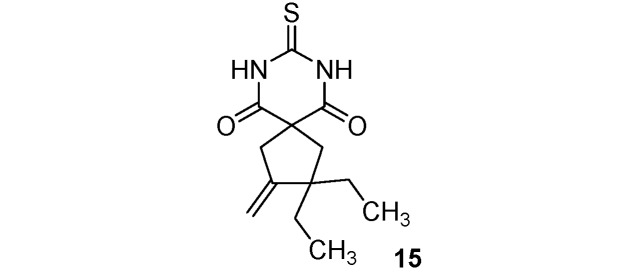	70%
8	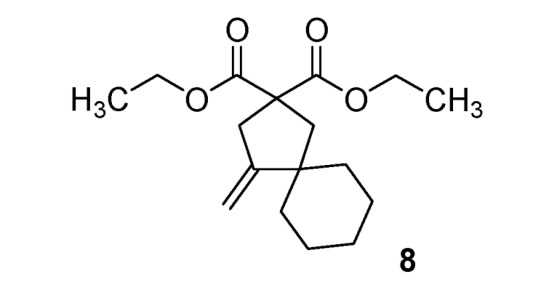	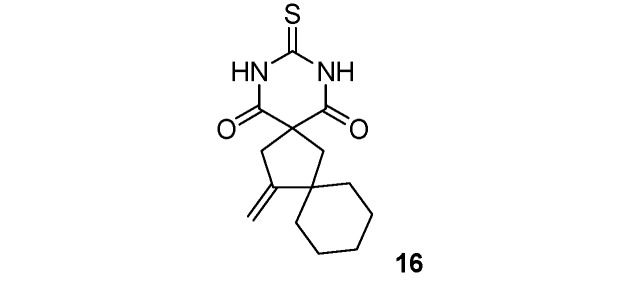	54%

**Scheme 3:**
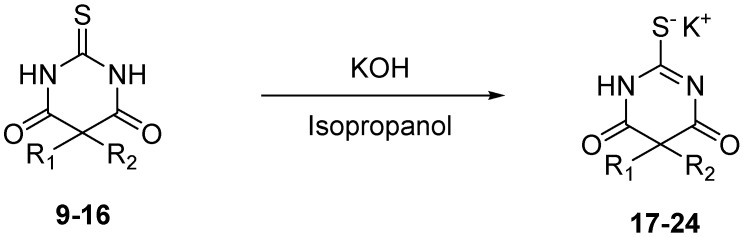
Thiobarbituric acid to thiobarbiturate salt formation.

Finally, in order to synthesize intravenous administrable thiobarbiturates, each thiobarbituric acid was turned into the corresponding salt with potassium hydroxide in isopropanol [37], as reported in Scheme 3.

## 3. Experimental

### 3.1. General

Microwave-assisted reactions were performed in a multimode microwave oven (ETHOS Synth Lab Station, Ethos start, Milestone Inc., Shelton, CT, USA). Melting points were determined with a B-540 Büchi melting point apparatus. ^1^H-NMR (200 MHz) and ^13^C-NMR (50 MHz) spectra were recorded on a Bruker ARX 200 spectrometer in CDCl_3_ or D_2_O at the Service interuniversitaire de RMN de la Faculté de Pharmacie de Marseille. The ^1^H-NMR chemical shifts are reported as parts per million downfield from tetramethylsilane (Me_4_Si), and the ^13^C-NMR chemical shifts were referenced to the solvent peaks: CDCl_3_ (76.9 ppm) or DMSO-*d_6_* (39.6 ppm). Absorptions are reported with the following notations: s, singlet; bs, broad singlet; d, doublet; t, triplet; q, quartet; m, a more complex multiplet or overlapping multiplets. Elemental analysis and mass spectra which were run on an API-QqToF mass spectrometer were carried out at the Spectropole de la Faculté des Sciences Saint-Jérôme site. Silica gel 60 (Merck, particle size 0.040–0.063 nm, 70–230 mesh ASTM) was used for flash column chromatography. TLC were performed on 5 cm × 10 cm aluminium plates coated with silica gel 60 F-254 (Merck, Gernsteim, Germany) in an appropriate solvent.

### 3.2. General Procedure for the Synthesis of Substituted Malonates **1–8**

*Method A*: A solution of manganese(III) acetate dihydrate (1.68 mmol, 0.45 g) in glacial acetic acid (55 mL) was heated under microwave irradiation (200 W, 80 °C) for 15 min, until dissolution. Then, the reaction mixture was cooled down to 60 °C, and a solution of malonate (3.99 mmol, 1 equiv.) and alkene (11.97 mmol, 3 equiv.) in glacial acetic acid (5 mL) was added. The mixture was heated under microwave irradiation (200 W, 80 °C) for 20 min. Then, the reaction mixture was cooled down to 60 °C once more, and a second portion of manganese(III) acetate dihydrate (1.68 mmol, 0.45 g) was added. The mixture was heated under microwave irradiation (200 W, 80 °C) for 20 min. The addition of manganese(III) acetate dihydrate (1.68 mmol, 0.45 g) was repeated three times under the same conditions every 20 min. successively. The reaction mixture was poured into cold water (100 mL), and extracted with chloroform (3 × 70 mL). The organic extracts were collected, washed with saturated aqueous NaHCO_3_ (3 × 50 mL) and brine (3 × 50 mL), dried over MgSO_4_, filtrated, and concentrated under vacuum. The crude product was purified by silica gel chromatography with ethyl acetate/petroleum ether (0.5/9.5) to give corresponding compounds **1**–**4**.

*Method B*: A solution of manganese(III) acetate dihydrate (8.38 mmol, 2.24 g, 2.1 equiv.) and copper(II) acetate monohydrate (3.99 mmol, 0.80 g, 1 equiv.) in glacial acetic acid (55 mL) was heated under microwave irradiation (200 W, 80 °C) for 15 min, until dissolution. Then, the reaction mixture was cooled down to 60 °C, and a solution of malonate (3.99 mmol, 1 equiv.) and alkene (7.98 mmol, 3 equiv.) in glacial acetic acid (5 mL) was added. The mixture was heated under microwave irradiation (200 W, 80 °C) for 60 min. The reaction mixture was poured into cold water (100 mL), and extracted with chloroform (3 × 70 mL). The organic extracts were collected, washed with saturated aqueous NaHCO_3_ (3 × 50 mL) and brine (3 × 50 mL), dried over MgSO_4_, filtrated, and concentrated under vacuum. The crude product was purified by silica gel chromatography with ethyl acetate/petroleum ether (0.5/9.5) to give corresponding compounds **1**–**8**.

*Diethyl 4*,*4-diethyl-3*,*4-dihydronaphthalene-2*,*2(1H)-dicarboxylate* (**1**). Colorless oil; yields: 49% (*method A*), 11% (*method B*); ^1^H-NMR (CDCl_3_) *δ*_H_ 0.77 (t, *J* = 7.3, 6H, 2CH_3_), 1.22 (t, *J = *7.2, 6H, 2CH_3_), 1.52–1.68 (m, 4H, 2CH_2_), 2.32 (s, 2H, CH_2_), 3.17 (s, 2H, CH_2_), 4.08–4.21 (m, 4H, 2CH_2_), 7.10–7.18 (m, 4H, 4CH). ^13^C-NMR (CDCl_3_) *δ*_C_ 8.3 (2CH_3_), 13.8 (2CH_3_), 33.1 (CH_2_), 33.3 (2CH_2_), 35.4 (CH_2_), 40.2 (C), 52.5 (C), 61.2 (2CH_2_), 125.5 (CH), 126.2 (CH), 126.5 (CH), 128.6 (CH), 134.2 (C), 141.5 (C), 172.9 (2C). HMRS (ESI): *m/z* calcd for C_20_H_28_O_4_[M+H^+^]: 333.2060. Found: 333.2061. 

*Diethyl 2-benzyl-2-(2-ethylbut-2-enyl)malonate* (**2a/2b**) (50:50 inseparable mixture of *Z/E* isomers). Colorless oil; yields: 10% (*method A*), 36% (*method B*); ^1^H-NMR (CDCl_3_) *δ*_H_ 0.89–0.99 (m, 3H, CH_3_), 1.12–1.22 (m, 6H, 2CH_3_), 1.54–1.64 (m, 3H, CH_3_), 1.93–2.04 (m, 2H, CH_2_), 2.63 and 2.80 (s, 2H, CH_2_), 3.24 and 3.26 (s, 2H, CH_2_), 4.03–4.15 (m, 4H, 2CH_2_), 5.26–5.42 (m, 1H, CH), 7.11–7.36 (m, 5H, 5CH). ^13^C-NMR (CDCl_3_) *δ*_C_ 12.7 (CH_3_), 12.8 and 13.2 (CH_3_), 13.8 and 13.9 (2CH_3_), 23.3 and 29.6 (CH_2_), 33.5 and 40.6 (CH_2_), 39.1 and 39.2 (CH_2_), 58.9 and 59.0 (C), 61.1 (2CH_2_), 122.2 and 123.0 (CH), 126.7 (CH), 128.0 (2CH), 130.1 (2CH), 130.2 (C), 136.8 and 137.3 (C), 171.5 and 171.6 (2C). HMRS (ESI): *m/z* calcd for C_20_H_28_O_4_ [M+H^+^]: 333.2060. Found: 333.2063.

*Diethyl 2'H-spiro[cyclohexane-1*,*1'-naphtalene]-3'*,*3'(4'H)-dicarboxylate* (**3**). [34] Colorless oil; yields: 52% (*method A*), 17% (*method B*); ^1^H-NMR (CDCl_3_) *δ*_H_ 1.22 (t, *J = *7.1, 6H, 2CH_3_), 1.47–1.80 (m, 10H, 5CH_2_), 2.46 (s, 2H, CH_2_), 3.19 (s, 2H, CH_2_), 4.14 (q, *J = *7.1, 2H, CH_2_), 4.15 (q, *J = *7.1, 2H, CH_2_), 7.10–7.23 (m, 3H, 3CH), 7.35–7.39 (m, 1H, 1CH). ^13^C-NMR (CDCl_3_) *δ*_C_ 13.9 (2CH_3_), 21.9 (2CH_2_), 25.9 (CH_2_), 34.9 (CH_2_), 35.6 (CH_2_), 36.8 (C), 39.6 (2CH_2_), 52.4 (C), 61.26 (2CH_2_), 125.8 (CH), 126.1 (CH), 126.5 (CH), 128.7 (CH), 133.4 (C), 144.0 (C), 171.8 (2C). Anal. Calcd for C_21_H_28_O_4_: C, 73.23; H, 8.19. Found: C, 73.40; H, 8.50. 

*Diethyl 2-benzyl-2-(cyclohexenylmethyl)malonate* (**4**). [34] Colorless oil; yields: 11% (*method A*), 31% (*method B*); ^1^H-NMR (CDCl_3_) *δ*_H_ 1.20 (t, *J = *7.1, 6H, 2CH_3_), 1.55–1.59 (m, 4H, 2CH_2_), 1.90–2.00 (m, 4H, 2CH_2_), 2.58 (s, 2H, CH_2_), 3.26 (s, 2H, CH_2_), 4.12 (q, *J = *7.1, 4H, 2CH_2_), 5.52 (s, 1H, 1CH), 7.11–7.24 (m, 5H, 5CH). ^13^C-NMR (CDCl_3_) *δ*_C_ 13.9 (2CH_3_), 22.1 (CH_2_), 23.0 (CH_2_), 25.5 (CH_2_), 29.2 (CH_2_), 39.0 (CH_2_), 41.4 (CH_2_), 58.7 (C), 61.0 (2CH_2_), 126.4 (CH), 126.7 (CH), 128.0 (2CH), 130.1 (2CH), 133.1 (C), 136.7 (C), 171.4 (2C). Anal. Calcd for C_21_H_28_O_4_: C, 73.23; H, 8.19. Found: C, 72.95; H, 8.35. 

*Diethyl 2-(2-ethylbut-2-enyl)-2-methylmalonate* (**5a/5b**) (50:50 inseparable mixture of *Z/E *isomers). Colorless oil; yields: 47% (*method B*); ^1^H-NMR (CDCl_3_) *δ*_H_ 0.81–0.93 (m, 3H, CH_3_), 1.14–1.21 (m, 6H, 2CH_3_), 1.27 (s, 3H, CH_3_), 1.48–1.53 (m, 3H, CH_3_), 1.65–1.96 (m, 2H, CH_2_), 2.57 and 2.71 (s, 2H, CH_2_), 4.04–4.15 (m; 4H, 2CH_2_), 5.13 and 5.34 (m, 1H, 1CH). ^13^C-NMR (CDCl_3_) *δ*_C_ 12.4 (CH_3_), 12.6 and 12.9 (CH_3_), 13.7 and 13.8 (CH_3_), 19.2 and 19.7 (CH_3_), 22.9 and 29.7 (CH_2_), 33.6 and 40.8 (CH_2_), 53.2 and 53.4 (C), 60.9 and 61.0 (2CH_2_), 122.4 and 123.4 (CH), 136.6 and 136.8 (C), 172.3 and 172.5 (2C). HMRS (ESI): *m/z* calcd for C_14_H_24_O_4_ [M+H^+^]: 257.1747. Found: 257.1743.

*Diethyl 2-(cyclohexenylmethyl)-2-methylmalonate* (**6**). Colorless oil; yields: 46% (*method B*); ^1^H-NMR (CDCl_3_) *δ*_H_ 1.23 (t, *J = *7.1 Hz, 6H, 2CH_3_), 1.34 (s, 3H, CH_3_), 1.44–1.58 (m, 4H, 2CH_2_), 1.73–2.03 (m, 4H, 2CH_2_), 2.58 (s, 2H, CH_2_), 4.15 (q, *J = *7.1, 2CH_2_), 5.43 (s, 1H, 1CH). ^13^C-NMR (CDCl_3_) *δ*_C_ 14.0 (2CH_3_), 19.9 (CH_3_), 22.0 (CH_2_), 22.9 (CH_2_), 25.4 (CH_2_), 29.2 (CH_2_), 43.7 (CH_2_), 53.3 (C), 61.1 (2CH_2_), 126.6 (CH), 132.9 (C), 172.6 (2C). HMRS (ESI): *m/z* calcd for C_15_H_24_O_4_ [M+H^+^]: 269.1747. Found: 269.1754.

*Diethyl 3*,*3-diethyl-4-methylenecyclopentane-1*,*1-dicarboxylate *(**7**). Colorless oil; yields: 26% (*method B*); ^1^H-NMR (CDCl_3_) *δ*_H_ 0.79 (t, *J = *7.3, 6H, 2CH_3_), 1.24 (t, *J = *7.1, 6H, 2CH_3_), 1.33–1.41 (m, 4H, 2CH_2_), 2.29 (s, 2H, CH_2_), 2.98–3.00 (m, 2H, CH_2_), 4.17 (q, *J = *7.1, 4H, 2CH_2_), 4.65 (bs, 1H, CH), 4.95 (bs, 1H, CH). ^13^C-NMR (CDCl_3_) *δ*_C _8.6 (2CH_3_), 14.0 (2CH_3_), 29.9 (2CH_2_), 41.8 (CH_2_), 43.3 (CH_2_), 48.5 (C), 57.3 (C), 61.4 (2CH_2_), 106.0 (CH_2_), 154.8 (C), 172.3 (2C). HMRS (ESI): *m/z* calcd for C_16_H_26_O_4_ [M+H^+^]: 283.1904. Found: 283.1906.

*Diethyl 4-methylenespir*o[4.5]d*ecane-2*,*2-dicarboxylate* (**8**). Colorless oil; yields: 68% (*method B*); ^1^H-NMR (CDCl_3_) *δ*_H_ 1.22 (t, *J = *7.2, 6H, 2CH_3_), 1.33–1.66 (m, 10H, 5CH_2_), 2.33 (s, 2H, CH_2_), 3.01 (bs, 2H, CH_2_), 4.15 (q, *J = *7.1, 4H, 2CH_2_), 4.77 (bs, 1H, CH), 4.87 (bs, 1H, CH). ^13^C-NMR (CDCl_3_) *δ*_C_ 13.9 (2CH_3_), 23.2 (2CH_2_), 25.8 (CH_2_), 38.0 (2CH_2_), 40.8 (CH_2_), 42.6 (CH_2_), 45.6 (C), 57.9 (C), 61.4 (2CH_2_), 104.6 (CH_2_), 158.4 (C), 172.1 (2C). HMRS (ESI): *m/z* calcd for C_17_H_26_O_4_[M+H^+^]: 295.1904. Found: 295.1903.

### 3.3. General Procedure for the Synthesis of Thiobarbituric Acids **9–16**

Thiourea (1.25 g, 16.38 mmol, 6 equiv.) was added to a solution of malonate **1–8** (2.73 mmol, 1 equiv.) in dry DMSO (3 mL). Then, a solution 1M of potassium *tert*-butoxide (0.67 g, 6.0 mmol, 2.2 equiv.) was added dropwise. The solution was stirred for 4 h under inert atmosphere and at rt (starting from malonates **1, 3, 7, 8**) or at 50 °C (starting from malonates **2, 4, 5, 6**). The solution was diluted with ethyl acetate (15 mL) and washed with a solution of 1 N hydrochloric acid. The layers were separated and the aqueous phase was extracted with ethyl acetate. The collected organic phase was washed with brine, dried over anhydrous Na_2_SO_4_, filtered and the solvent was removed *in vacuo*. The residue was purified with column chromatography (CH_2_Cl_2_/petroleum ether, 8:2), affording the corresponding thiobarbituric acids **9–16**.

*4*,*4-Diethyl-2'-thioxo-3*,*4-dihydro-1H*,*2'H-spiro[naphthalene-2*,*5'-pyrimidine]-4'*,*6'(1'H*,*3'H)-dione* (**9**). White solid; m.p. 151 °C (cyclohexane); yields: 53% ^1^H-NMR (CDCl_3_) *δ*_H_ 0.76 (t, *J = *7.4, 6H, 2CH_3_), 1.67–1.80 (m, 4H, 2CH_2_), 2.23 (s, 2H, CH_2_), 3.28 (s, 2H, CH_2_), 7.12–7.36 (m, 4H, 4CH), 8.99 (bs, 2H). ^13^C-NMR (CDCl_3_) *δ*_C_ 8.4 (2CH_3_), 31.5 (2CH_2_), 34.3 (CH_2_), 38.2 (CH_2_), 52.2 (C), 53.4 (C), 126.0 (CH), 126.2 (CH), 126.8 (CH), 128.5 (CH), 132.4 (C), 140.9 (C), 170.4 (2C), 176.0 (C). HMRS (ESI): *m/z* calcd for C_17_H_20_N_2_O_2_S [M+H^+^]: 317.1318. Found: 317.1317.

*5-Benzyl-5-(2-ethylbut-2-enyl)-2-thioxo-dihydropyrimidine-4*,*6(1H*,*5H)-dione *(**10a/10b**) (50:50 inseparable mixture of *Z/E *isomers). White solid; m.p. 182 °C (cyclohexane); yields: 46% ^1^H-NMR (CDCl_3_) *δ*_H_ 0.90–0.99 (m, 3H, CH_3_), 1.53–1.66 (m, 3H, CH_3_), 1.85–2.02 (m, 2H, CH_2_), 2.87 and 3.00 (s, 2H, CH_2_), 3.30 and 3.38 (s, 2H, CH_2_), 5.19–5.30 and 5.41–5.52 (m, 1H, CH), 7.07–7.24 (m, 5H, 5CH), 8.84 (bs, 2H). ^13^C-NMR (CDCl_3_) *δ*_C_ 12.6 and 13.0 (CH_3_), 13.4 and 13.7 (CH_3_), 23.4 and 29.9 (CH_2_), 39.1 and 44.9 (CH_2_), 45.0 and 45.2 (CH_2_), 58.0 and 59.0 (C), 124.6 and 124.8 (CH), 127.9 (CH), 128.9 (2CH), 129.5 and 129.6 (2CH), 134.2 and 134.3 (C), 134.7 and 135.7 (C), 169.6 (2C), 175.3 (C). *m/z* calcd for C_17_H_20_N_2_O_2_S [M+H^+^]: 317.1318. Found: 317.1323.

*2"-Thioxo-2"H*,*4'H-dispiro[cyclohexane-1*,*1'-naphtalene-3'*,*5"-pyrimidine]-4"*,*6"(1"H,3"H)-dione *(**11**). White solid; m.p. 200–202 °C (ethyl alcohol); yields: 64% ^1^H-NMR (CDCl_3_) *δ*_H_ 1.49–1.84 (m, 10H, 5CH_2_), 2.35 (s, 2H, CH_2_), 3.31 (s, 2H, CH_2_), 7.12–7.41 (m, 4H, 4CH), 9.33 (bs, 2H, 2NH). ^13^C-NMR (CDCl_3_) *δ*_C_ 22.0 (2CH_2_), 25.7 (CH_2_), 33.6 (CH_2_), 37.8 (C), 38.1 (2CH_2_), 38.3 (CH_2_), 52.2 (C), 125.1 (CH), 126.1 (CH), 127.2 (CH), 128.5 (CH), 132.1 (C), 143.8 (C), 170.2 (2C), 176.0 (C). HMRS (ESI): *m/z* calcd for C_18_H_20_N_2_O_2_S [M+H^+^]: 329.1318. Found: 329.1317.

*5-Benzyl-5-(cyclohexenylmethyl)-2-thioxo-dihydropyrimidine-4*,*6(1H*,*5H)-dione* (**12**). Colorless oil; yields: 88% ^1^H-NMR (CDCl_3_) *δ*_H_ 1.35–2.04 (m, 8H, 4CH_2_), 2.82 (s, 2H, CH_2_), 3.31 (s, 2H, CH_2_), 5.50 (s, 1H, 1CH), 7.13–7.26 (m, 5H, 5CH), 8.98 (bs, 2H, 2NH). ^13^C-NMR (CDCl_3_) *δ*_C_ 21.9 (CH_2_), 22.8 (CH_2_), 23.6 (CH_2_), 29.8 (CH_2_), 44.5 (CH_2_), 47.6 (CH_2_), 58.9 (C), 127.7 (CH), 127.8 (CH), 128.8 (2CH), 129.5 (2CH), 131.5 (C), 134.3 (C), 169.7 (2C), 175.4 (C). HMRS (ESI): *m/z* calcd for C_18_H_20_N_2_O_2_S [M+NH_4_^+^]: 346.1584. Found: 346.1579. 

*5-(2-Ethylbut-2-enyl)-5-methyl-2-thioxo-dihydropyrimidine-4*,*6(1H*,*5H)-dione* (**13a/13b**) (50:50 inseparable mixture of *Z/E* isomers). Colorless oil; yields: 75% ^1^H-NMR (CDCl_3_) *δ*_H_ 0.87–0.97 (m, 3H, CH_3_), 1.54–1.61 (m, 3H, CH_3_), 1.57 (s, 3H, CH_3_), 1.80–2.01 (m, 2H, CH_2_), 2.70 and 2.82 (s, 2H, CH_2_), 5.18 and 5.47 (m, 1H, CH), 9.05 (bs, 2H, 2NH). ^13^C-NMR (CDCl_3_) *δ*_C_ 12.6 and 13.0 (CH_3_), 13.3 and 13.9 (CH_3_), 23.1 and 23.3 (CH_3_), 23.5 and 29.9 (CH_2_), 40.4 and 46.2 (CH_2_), 51.0 and 51.9 (C), 124.5 and 125.0 (CH), 134.8 and 135.9 (C), 170.5 and 170.6 (2C), 176.0 (C). Anal. Calcd for C_11_H_16_N_2_O_2_S: C, 54.98; H, 6.71; N, 11.66. Found: C, 55.15; H, 6.86; N, 11.63.

*5-(Cyclohexenylmethyl)-5-methyl-2-thioxo-dihydropyrimidine-4*,*6(1H*,*5H)-dione* (**14**). White solid; m.p. 160–164 °C (ethyl alcohol); yields: 90% ^1^H-NMR (CDCl_3_) *δ*_H_ 1.37–1.52 (m, 4H, 2CH_2_), 1.57 (s, 3H, CH_3_), 1.76–1.98 (m, 4H, 2CH_2_), 2.65 (s, 2H, CH_2_), 5.44 (s, 1H, 1CH), 9.61 (bs, 2H, 2NH). ^13^C-NMR (CDCl_3_) *δ*_C_ 21.8 (CH_2_), 22.8 (CH_2_), 23.0 (CH_3_), 25.4 (CH_2_), 29.7 (CH_2_), 48.5 (CH_2_), 51.8 (C), 127.5 (CH), 131.6 (C), 170.9 (2C), 176.2 (C). HMRS (ESI): *m/z* calcd for C_12_H_16_N_2_O_2_S [M+H^+^]: 253.1005. Found: 253.1007.

*2*,*2-Diethyl-3-methylene-8-thioxo-7*,*9-diazaspiro[4.5]decane-6*,*10-dione* (**15**). White solid; m.p. 194–196 °C (cyclohexane); yields: 70% ^1^H-NMR (CDCl_3_) *δ*_H_ 0.83 (t, *J = *7.4, 6H, 2CH_3_), 1.43–1.70 (m, 4H, 2CH_2_), 2.27 (s, 2H, CH_2_), 3.03 (bs, 2H, CH_2_), 4.77 (bs, 1H, CH), 5.01 (bs, 1H, CH), 8.96 (bs, 2H, 2NH). ^13^C-NMR (CDCl_3_) *δ*_C_ 8.7 (2CH_3_), 29.0 (2CH_2_), 44.4 (CH_2_), 47.2 (CH_2_), 49.9 (C), 54.3 (C), 107.4 (CH_2_), 153.4 (C), 170.7 (2C), 176.1 (C). HMRS (ESI): *m/z* calcd for C_13_H_18_N_2_O_2_S [M+NH_4_^+^]: 284.1427. Found: 284.1434.

*14-Methylene-3-thioxo-2*,*4-diazadispiro[5.1.5.2]pentadecane-1*,*5-dione* (**16**). White solid; m.p. 177 °C (isopropanol); yields: 54% ^1^H-NMR (CDCl_3_) *δ*_H_ 1.22–1.47 (m, 6H, 2CH_3_), 1.66–1.77 (m, 4H, 2CH_2_), 2.33 (s, 2H, CH_2_), 3.06 (s, 2H, CH_2_), 4.89–4.93 (m, 2H, CH_2_), 9.09 (bs, 2H, 2NH). ^13^C-NMR (CDCl_3_) *δ*_C_ 23.2 (2CH_2_), 25.7 (CH_2_), 37.5 (2CH_2_), 44.0 (CH_2_), 45.2 (CH_2_), 46.8 (C), 55.0 (C), 105.4 (CH_2_), 157.4 (C), 170.7 (2C), 176.2 (C). HMRS (ESI): *m/z* calcd for C_14_H_18_N_2_O_2_S [M+NH_4_^+^]: 296.1427. Found: 296.1422.

### 3.4. General Procedure for Salification of Barbituric Acids to Barbiturate Potassium Salts **17–24**

A suspension of potassium hydroxide (0.02 g, 0.36 mmol, 1 equiv.) in isopropanol (5 mL) was stirred under inert atmosphere. The corresponding barbituric acid **9–16** (0.36 mmol, 1 equiv.) was added, and reaction was monitored by TLC until the barbituric acid disappeared. Isopropanol was removed *in vacuo*, and corresponding barbiturates **17–24** were obtained without further purification. 

*Potassium 4*,*4-diethyl-4'*,*6'-dioxo-1'*,*3*,*4*,*6'-tetrahydro-1H*,*4'H-spiro[naphthalene-2*,*5'-pyrimidine]-2'-thiolate* (**17**). White solid; m.p. 161–163 °C (isopropanol); yields: 77%; ^1^H-NMR (D_2_O) *δ*_H_ 0.72 (s, 3H, CH_3_), 0.99 (s, 3H, CH_3_), 1.42–1.79 (m, 4H, 2CH_2_), 2.38 (d, *J* = 15.4, 1H, CH_2_), 2.53 (d, *J* = 15.4, 1H, CH_2_), 3.13 (d, *J* = 16.4, 1H, CH_2_), 3.40 (d, *J* = 16.4, 1H, CH_2_), 7.35–7.41 (m, 4H, 4CH). ^13^C-NMR (D_2_O) *δ*_C_ 8.4 (CH_3_), 8.5 (CH_3_), 32.9 (CH_2_), 35.1 (CH_2_), 35.2 (CH_2_), 35.8 (CH_2_), 41.2 (C), 57.2 (C), 126.5 (CH), 127.0 (CH), 127.9 (CH), 129.1 (CH), 136.2 (C), 142.8 (C), 177.0 (2C), 178.9 (C). HMRS (ESI): *m/z* calcd for C_17_H_19_N_2_O_2_S^−^ M: 315.1173. Found: 315.1183. 

*Potassium 5-benzyl-5-[2-ethylbut-2-en-1-yl]-4*,*6-thioxo-1*,*4*,*5*,*6-tetrahydropyrimidine-2-thiolate* (**18a/** **18b**) (50:50 inseparable mixture of *Z/E *isomers). White solid; m.p. 142–144 °C (isopropanol); yields: 78%; ^1^H-NMR (D_2_O) *δ*_H_ 0.98–1.05 (m, 3H, CH_3_), 1.61–1.73 (m, 3H, CH_2_), 1.93–2.12 (m, 2H, CH_2_), 2.89 and 3.01 (s, 2H, CH_2_), 3.28 and 3.38 (s, 2H, CH_2_), 5.11 and 5.51 (bs, 1H, 1CH), 7.22–7.39 (m, 5H, 5CH). ^13^C-NMR (D_2_O) *δ*_C_ 12.6 and 12.7 (CH_3_), 13.2 and 13.3 (CH_3_), 23.6 and 29.8 (CH_2_), 39.3 and 44.8 (CH_2_), 45.0 and 45.9 (CH_2_), 57.6 (C), 122.7 and 123.8 (CH), 128.0 (CH), 129.1 (2CH), 129.9 (2CH), 135.9 (C), 138.0 (C), 172.9 (C), 179.6 (2C). HMRS (ESI): *m/z* calcd for C_17_H_19_N_2_O_2_S^−^ M: 315.1173. Found: 315.1180. 

*Potassium 4"*,*6"-dioxo-1"*,*6"-dihydro-4'H*,*4"H-dispiro[cyclohexane-1*,*1'-naphtalene-3'*,*5"-pyrimidine]-2"-thiolate* (**19**). White solid; m.p. 216–218 °C (isopropanol); yields: 70%; ^1^H-NMR (D_2_O) *δ*_H_ 1.38–2.25 (m, 10H, 5CH_2_), 2.40 (bs, 1H, CH_2_), 3.08–3.68 (m, 3H, CH_2_), 7.40–7.58 (m, 3H, 3CH), 7.72–7.78 (m, 1H, 1CH). ^13^C-NMR (D_2_O) *δ*_C_ 22.1 (CH_2_), 22.4 (CH_2_), 26.0 (CH_2_), 35.8 (CH_2_), 37.6 (CH_2_), 37.7 (C), 38.2 (CH_2_), 42.0 (CH_2_), 56.9 (C), 126.8 (CH), 127.2 (CH), 127.3 (CH), 129.3 (CH), 135.3 (C), 144.7 (C), 176.5 (C), 178.8 (C), 181.5 (C). HMRS (ESI): *m/z* calcd for C_18_H_19_N_2_O_2_S^−^ M: 327.1173. Found: 327.1184.

*Potassium 5-benzyl-5-(cyclohex-1-en-1-ylmethyl)-4*,*6-dioxo-1*,*4*,*5*,*6-tetrahydropyrimidine-2-thiolate* (**20**). White solid; m.p. 143 °C (isopropanol); yields: 84% ^1^H-NMR (D_2_O) *δ*_H_ 1.36–1.60 (m, 4H, 2CH_2_), 1.74–2.00 (m, 4H, 2CH_2_), 2.70 (s, 2H, CH_2_), 3.18 (s, 2H, CH_2_), 5.39 (s, 1H, 1CH), 7.06–7.11 (m, 2H, 2CH), 7.26–7.30 (m, 3H, 3CH). ^13^C-NMR (D_2_O) *δ*_C_ 22.3 (CH_2_), 23.3 (CH_2_), 25.7 (CH_2_), 29.8 (CH_2_), 45.5 (CH_2_), 47.6 (CH_2_), 57.3 (C), 126.2 (CH), 127.8 (CH), 129.1 (2CH), 129.9 (2CH), 134.0 (C), 136.6 (C), 181.5 (2C), 192.6 (C). HMRS (ESI): *m/z* calcd for C_18_H_19_N_2_O_2_S^−^ M: 327.1173. Found: 327.1173. 

*Potassium 5-[2-ethylbut-2-en-1-yl]-5-methyl-4*,*6-dioxo-1*,*4*,*5*,*6-tetrahydropyrimidine-2-thiolate *(**21a/** **21b**) (50:50 inseparable mixture of *Z/E *isomers). White solid; m.p. 174–176 °C (isopropanol); yields: 28% ^1^H-NMR (D_2_O) *δ*_H_ 0.82–0.98 (m, 3H, CH_3_), 1.32–1.42 (m, 3H, CH_3_), 1.49–1.56 (m, 3H, CH_3_), 1.76–2.05 (m, 2H, CH_2_), 2.54–2.69 (m, 2H, CH_2_), 5.01 and 5.45 (bs, 1H, 1CH). ^13^C-NMR (D_2_O) *δ*_C_ 12.8 and 13.0 (CH_3_), 13.2 and 14.0 (CH_3_), 21.0 and 22.7 (CH_3_), 23.7 and 30.2 (CH_2_), 38.5 and 44.8 (CH_2_), 56.7 and 57.0 (C), 123.1 and 123.8 (CH), 138.3 and 139.1 (C), 177.8 and 177.9 (C), 180.0 and 180.1 (C), 181.5 and 181.6 (C). HMRS (ESI): *m/z* calcd for C_11_H_15_N_2_O_2_S^−^ M: 239.0860. Found: 239.0857.

*Potassium 5-(cyclohex-1-en-1-ylmethyl)-5-methyl-4*,*6-dioxo-1*,*4*,*5*,*6-tetrahydropyrimidine-2-thiolate *(**22**). White solid; m.p. 177 °C (isopropanol); yields: 69% ^1^H-NMR (D_2_O) *δ*_H_ 1.47 (s, 3H, CH_3_), 1.45–1.61 (m, 4H, 2CH_2_), 1.84–2.09 (m, 4H, 2CH_2_), 2.55 (s, 2H, CH_2_), 5.41 (s, 1H, 1CH). ^13^C-NMR (D_2_O) *δ*_C_ 22.4 (CH_2_), 22.5 (CH_3_), 23.3 (CH_2_), 25.7 (CH_2_), 29.6 (CH_2_), 47.4 (CH_2_), 56.8 (C), 126.7 (CH), 134.9 (C), 177.9 (2C), 181.6 (C). HMRS (ESI): *m/z* calcd for C_12_H_15_N_2_O_2_S^−^ M: 251.0860. Found: 251.0859.

*Potassium 2*,*2-diethyl-3-methylene-6*,*10-dioxo-7*,*9-diazaspiro[4.5]dec-7-ene-8-thiolate* (**23**). White solid; decomp. 270 °C (isopropanol); yields: 88% ^1^H-NMR (D_2_O) *δ*_H_ 0.72–0.83 (m, 6H, 2CH_3_), 1.14–1.53 (m, 4H, 2CH_2_), 2.27 (s, 2H, CH_2_), 2.84 (d, *J* = 16.3, 1H, CH_2_), 3.04 (d, *J* = 16.3, 1H, CH_2_), 4.72 (bs, 1H, CH), 5.01 (bs, 1H, CH). ^13^C-NMR (D_2_O) *δ*_C_ 8.6 (CH_3_), 8.7 (CH_3_), 30.5 (CH_2_), 31.0 (CH_2_), 41.7 (CH_2_), 45.0 (CH_2_), 49.1 (C), 62.5 (C), 105.8 (CH_2_), 157.2 (C), 176.7 (C), 179.1 (C), 182.1 (C). HMRS (ESI): *m/z* calcd for C_13_H_17_N_2_O_2_S^−^ M: 265.1016. Found: 265.1025.

*Potassium 14-methylene-1*,*5-dioxo-2*,*4-diazaspiro[5.1.5.2]pentadec-2-ene-3-thiolate* (**24**). White solid; m.p. 174–176 °C (isopropanol); yields: 53% ^1^H-NMR (D_2_O) *δ*_H_ 1.13–1.65 (m, 10H, 5CH_2_), 2.25 (d, *J = *14.0, 1H, CH_2_), 2.40 (d, *J = *14.0, 1H, CH_2_), 2.88 (d, *J = *16.4, 1H, CH_2_), 3.04 (d, *J = *16.4, 1H, CH_2_), 4.86 (bs, CH), 4.96 (bs, CH). ^13^C-NMR (D_2_O) *δ*_C_ 22.8 (CH_2_), 22.9 (CH_2_), 37.6 (CH_2_), 38.6 (CH_2_), 40.1 (CH_2_), 44.0 (CH_2_), 45.7 (C), 62.3 (C), 104.0 (CH_2_), 160.7 (C), 175.9 (C), 178.3 (C). 1C not observed in these conditions. HMRS (ESI): *m/z* calcd for C_14_H_17_N_2_O_2_S^−^ M: 277.1016. Found: 277.1009.

## 4. Conclusions

We have synthesized eight new functionalized thiobarbiturates by a three steps synthesis, thanks to Mn(OAc)_3_ radical reactivity. This methodology allows *C*-functionalization of barbituric acid with a wide variety of scaffolds, such as aromatic, aliphatic and spirocyclic moieties. Derivatives thus obtained could be tested for their anesthetic potentialities, but also for targeting anticonvulsive leads.

## References

[1] Snider B.B. (1996). Manganese(III)-based oxidative free-radical cyclizations. Chem. Rev..

[2] Demir A.S., Emrullahoglu M. (2007). Manganese(III) acetate: A versatile reagent in organic chemistry. Curr. Org. Synth..

[3] Dombroski M.A., Snider B.B. (1992). Manganese(III)-Based oxidative free-radical. Cyclizations of γ,γ-*bis*(allylic) acetoacetates. Tetrahedron.

[4] Kates S.A., Dombroski M.A., Snider B.B. (1990). Manganese(III)-based oxidative free-radical cyclization of unsaturated beta-keto esters, 1,3-diketones, and malonate diesters. J. Org. Chem..

[5] Chuang C.-P., Tsai A.-I. (2007). A novel oxidative free radical reaction between 2-amino-1,4-benzoquinones and benzoylacetonitriles. Tetrahedron.

[6] Logoglu E., Yilmaz M., Katircioglu H., Yakut M., Mercan S. (2010). Synthesis and biological activity studies of furan derivatives. Med. Chem. Res..

[7] Curti C., Crozet M.D., Vanelle P. (2009). Microwave-assisted manganese(III) acetate based oxidative cyclizations of alkenes with β-ketosulfones. Tetrahedron.

[8] Bouhlel A., Curti C., Dumètre A., Laget M., Crozet M.D., Azas N., Vanelle P. (2010). Synthesis and evaluation of original amidoximes as antileishmanial agents. Bioorg. Med. Chem..

[9] Paloque L., Bouhlel A., Curti C., Dumètre A., Verhaeghe P., Azas N., Vanelle P. (2011). Synthesis and evaluation of monoamidoxime derivatives: Toward new antileishmanial compounds. Eur. J. Med. Chem..

[10] Biltz H., Witteck H. (1921). Alkylated and acylated barbituric acids. Ber. Dtsch. Chem. Ges..

[11] Clark-Lewis J.W., Thompson M.J. (1959). Preparation of 1,3-dimethylbarbituric acid and formation of 5-ethoxycarbonylacetyl-1,3-dimethylbarbituric acid. J. Chem. Soc..

[12] Nguyen V., Nishino H. (2004). Novel synthesis of dihydropyrans and 2,8-dioxabicylo[3.3.0]oct-3-enes using Mn(III)-based oxidative cyclization. Tetrahedron Lett..

[13] Tsubusaki T., Nishino H. (2009). Manganese(III)-mediated facile synthesis of 3,4-dihydro-2(1H)-quinolinones: Selectivity of the 6-endo and 5-exo cyclization. Tetrahedron.

[14] Delmas F., Gasquet M., Timon-David P., Madadi N., Vanelle P., Vaille A., Maldonado J. (1993). Synthesis and *in vitro* anti-protozoan activity of new 5-nitrothiophene oxime ether derivatives. Eur. J. Med. Chem..

[15] Baraldi P.G., El-Kashef H., Farghaly A.R., Vanelle P., Fruttarolo F. (2004). Synthesis of new pyrazolo[4,3-e]-1,2,4-triazolo[1,5-c]pyrimidines and related heterocycles. Tetrahedron.

[16] Boufatah N., Gellis A., Maldonado J., Vanelle P. (2004). Efficient microwave-assisted synthesis of new sulfonylbenzimidazole-4,7-diones: Heterocyclic quinones with potential antitumor activity. Tetrahedron.

[17] Verhaeghe P., Azas N., Gasquet M., Hutter S., Ducros C., Laget M., Rault S., Rathelot P., Vanelle P. (2008). Synthesis and antiplasmodial activity of new 4-aryl-2-trichloromethylquinazolines. Bioorg. Med. Chem. Lett..

[18] Kabri Y., Gellis A., Vanelle P. (2009). Microwave-assisted synthesis in aqueous medium of new quinazoline derivatives as anticancer agent precursors. Green Chem..

[19] Smith M.C., Riskin B.J. (1991). The clinical use of barbiturates in neurological disorders. Drugs.

[20] Nagel S., von Heinemann P., Heiland S., Koziol J., Gardner H., Wagner S. (2011). Selective MMP-inhibition with Ro 28-2653 in acute experimental stroke—A magnetic resonance imaging efficacy study. Brain Res..

[21] Wang J., Medina C., Radomski M.W., Gilmer J.F. (2011). N-substituted homopiperazine barbiturates as gelatinase inhibitors. Bioorg. Med. Chem..

[22] Vijaya L.S., Thirupathi R.Y., Suresh K.B., Narsimha R.P., Crooks P.A., Rajitha B. (2011). Synthesis and evaluation of chromenyl barbiturates and thiobarbiturates as potential antitubercular agents. Bioorg. Med. Chem. Lett..

[23] Al-Najjar B.O., Wahab H.A., Muhammad T.S.T., Shu-Chien A.C., Noruddin N.A.A., Taha M.O. (2011). Discovery of new nanomolar peroxisome proliferator-activated receptor γ activators via elaborate ligand-based modeling. Eur. J. Med. Chem..

[24] Ma L., Li S., Zheng H., Chen J., Lin L., Ye X., Chen Z., Xu Q., Chen T., Yang J. (2011). Synthesis and biological activity of novel barbituric and thiobarbituric acid derivatives against non-alcoholic fatty liver disease. Eur. J. Med. Chem..

[25] Zheng H., Li S., Ma L., Cheng L., Deng C., Chen Z., Xie C., Xiang M., Jiang W., Chen L. (2011). A novel agonist of PPAR-γ based on barbituric acid alleviates the development of non-alcoholic fatty liver disease by regulating adipocytokine expression and preventing insulin resistance. Eur. J. Pharmacol..

[26] Gruber P., Rechfeld F., Kirchmair J., Hauser N., Boehler M., Garczarczyk D., Langer T., Hofmann J. (2011). Barbituric acid derivative BAS 02104951 inhibits PKCε, PKCη, PKCε/RACK2 interaction, Elk-1 phosphorylation in HeLa and PKCε and η translocation in PC3 cells following TPA-induction. J. Biochem..

[27] Soine W., Foye W.O., Lemke T.L., Williams D.A. (2007). Sedative-Hypnotics. Foye’s Principles of Medicinal Chemistry.

[28] Kepczyńska E., Obłoza E., Stasiewicz-Urban A., Bojarski J., Pyka A. (2007). Lipophilicity of thiobarbiturates determined by TLC. Acta Pol. Pharm..

[29] Toon S., Rowland M. (1983). Structure-pharmacokinetic relationships among the barbiturates in the rat. J. Pharmacol. Exp. Ther..

[30] Snider B.B., Patricia J.J., Kates S.A. (1988). Mechanism of manganese(III)-based. Oxidation of β-keto esters. J. Org. Chem..

[31] Citterio A., Sebastiano R., Marion A. (1991). Synthesis of substituted tetrahydronaphthalenes by manganese(III), cerium(IV), and iron(III) oxidation of substituted diethyl alpha-benzylmalonates in the presence of olefins. J. Org. Chem..

[32] Santi R., Bergamini F., Citterio A., Sebastiano R., Nicolini M. (1992). Reactivity of malonyl radicals. Synthesis of substituted dihydronaphthalenes by manganese(III) oxidation of diethyl alpha-benzylmalonate in the presence of alkynes. J. Org. Chem..

[33] Bergamini F., Citterio A., Gatti N., Nicolini M., Santi R., Sebastiano R. (1993). Metal-induced electrochemical oxidation of diethyl benzylmalonates in the presence of alkenes and alkynes. Synthesis of substituted tetrahydro- and dihydronaphthalenes. J. Chem. Res. (S).

[34] Bouhlel A., Curti C., Vanelle P. (2012). Access to original spirocyclic derivatives via inter- or intramolecular reaction mediated by manganese(III) acetate. Tetrahedron.

[35] Snider B.B., Buckman B.O. (1989). Manganese(III) based oxidative free-radical annulations. Tetrahedron.

[36] Jagodzinska M., Huguenot F., Candiani G., Zanda M. (2009). Assessing the bioisosterism of the trifluoromethyl group with a protease probe. Chem. Med. Chem..

[37] Sun T., Watson S., Manchanda R. (2010). Disclosed are new phenobarbital salts, methods of preparation, and uses thereof. U.S. Patent 0035904, 11 February 2010. Chem. Abstr..

